# Comparative genomics analysis of *Streptococcus agalactiae* reveals that isolates from cultured tilapia in China are closely related to the human strain A909

**DOI:** 10.1186/1471-2164-14-775

**Published:** 2013-11-11

**Authors:** Guangjin Liu, Wei Zhang, Chengping Lu

**Affiliations:** 1 Key Laboratory of Animal Bacteriology, Ministry of Agriculture, Nanjing Agricultural University, Weigang No.1, Nanjing, Jiangsu 210095, China

**Keywords:** *Streptococcus agalactiae*, Tilapia, Pan-genome, China

## Abstract

**Background:**

*Streptococcus agalactiae*, also referred to as Group B Streptococcus (GBS), is a frequent resident of the rectovaginal tract in humans, and a major cause of neonatal infection. In addition, *S. agalactiae* is a known fish pathogen, which compromises food safety and represents a zoonotic hazard. The complete genome sequence of the piscine *S. agalactiae* isolate GD201008-001 was compared with 14 other piscine, human and bovine strains to explore their virulence determinants, evolutionary relationships and the genetic basis of host tropism in *S. agalactiae*.

**Results:**

The pan-genome of *S. agalactiae* is open and its size increases with the addition of newly sequenced genomes. The core genes shared by all isolates account for 50 ~ 70% of any single genome. The Chinese piscine isolates GD201008-001 and ZQ0910 are phylogenetically distinct from the Latin American piscine isolates SA20-06 and STIR-CD-17, but are closely related to the human strain A909, in the context of the clustered regularly interspaced short palindromic repeats (CRISPRs), prophage, virulence-associated genes and phylogenetic relationships. We identified a unique 10 kb gene locus in Chinese piscine strains.

**Conclusions:**

Isolates from cultured tilapia in China have a close genomic relationship with the human strain A909. Our findings provide insight into the pathogenesis and host-associated genome content of piscine *S. agalactiae* isolated in China.

## Background

*Streptococcus agalactiae*, also referred to as Group B Streptococcus (GBS), is a Gram-positive, β-hemolytic, chain-forming coccus that comprises vaginal flora in 25% of healthy women [1]. It has been recognized as one of the major causes of pneumonia, meningitis in neonates [2], mastitis in cows [3], and meningoencephalitis in fish [4,5]. Piscine GBS affects a variety of wild and cultured fish species worldwide, particularly in tilapia, and causes high mortality, resulting in large economic losses on fish farms [6]. Since 2009, large-scale *S. agalactiae* infectious outbreaks have occurred continuously in tilapia farms in China [7]. The GBS isolate GD201008-001, a highly virulent strain isolated from moribund cultured tilapia with meningoencephalitis in Guangdong province of China in 2010, belongs to serotype Ia, multilocus sequence type 7 (ST7) [8]. This lineage serotype Ia, ST-7 GBS is associated with bloodstream infections in human neonates and adults [9,10]. The pathogenicity of human Ia, ST-7 isolates toward tilapia is well-established [11]. Consumption of tilapia has been associated with an increased risk of *S. agalactiae* serotype Ia and Ib colonization in humans [12].

To date, five complete genome sequences and 10 draft genome sequences of *S. agalactiae* have been made publicly available, including nine isolates of human origin, four of fish origin and two of bovine origin. In this study, we aimed to perform comparative analyses of these sequence data to identify virulence determinants, evolutionary relationships and the genetic basis associated with host tropism in *Streptococcus agalactiae*.

## Results and discussion

### Characteristics of the 15 *S. agalactiae* genomes

The characteristics of the strains and their genomes utilized in this analysis are shown in Table 1. The strains represent different host tropisms (human, fish, bovine), collected from China, North America, Latin America and Europe. The genome sizes range between 1.81 to 2.46 Mbp (a 15.9% difference), with a mean size of 2.11 Mbp. For piscine *S. agalactiae*, two Chinese isolates, GD201008-001 and ZQ0910, share the same genome size (2.1 Mbp), G + C content (35.6%) and lineage serotype (Ia, ST-7) with the human isolate A909. SA20-06 and STIR-CD-17, isolated from fish in Latin-America, have the smallest genomes, only 1.8 Mb encoding 1,700 proteins and belong to Ib, ST-553 and Ib, ST-260, respectively. The serotype Ib, ST-260 isolates belong to a fish-associated clonal complex that has never been reported from humans [13].

**Table 1:** Sequenced strains and available genomes used in this study

**Strain**	**Sero type**	**MLST types**	**NCBI accession NO.**	**Status**	**Size (bp)**	**GC%**	**Protein**	**Isolate host**	**Origin**
*S. agalactiae* GD201008-001	Ia	ST-7	NC_018646	Complete	2,063,112	35.6	1,964	Fish	China
*S. agalactiae* SA20-06	Ib	ST-553	NC_019048	Complete	1,820,886	35.6	1,710	Fish	Brazil
*S. agalactiae* ZQ0910	Ia	ST-7	NZ_AKAP00000000	Scaffolds	2,008,809	35.4	1,970	Fish	China
*S. agalactiae* STIR-CD-17	Ib	ST-260	NZ_ALXB00000000	Scaffolds	1,805,303	35	1,698	Fish	Honduras
*S. agalactiae* A909	Ia	ST-7	NC_007432	Complete	2,127,839	35.6	1,996	Human	USA
*S. agalactiae* NEM316	III	ST-23	NC_004368	Complete	2,211,485	35.6	2,094	Human	France
*S. agalactiae* 2603 V/R	V	ST-110	NC_004116	Complete	2,160,267	35.6	2,124	Human	USA
*S. agalactiae* 18RS21	II	ST-19	NZ_AAJO00000000	Scaffolds	2,192,158	36.9	2,146	Human	USA
*S. agalactiae* 515	Ia	ST-23	NZ_AAJP00000000	Scaffolds	2,088,029	35.3	2,275	Human	USA
*S. agalactiae* CJB111	V	ST-1	NZ_AAJQ00000000	Scaffolds	2,104,864	35.6	2,197	Human	USA
*S. agalactiae* COH1	III	ST-17	NZ_AAJR00000000	Scaffolds	2,204,946	35.5	2,376	Human	USA
*S. agalactiae* GB00112	III	ST-17	NZ_AKXO00000000	Scaffolds	2,033,051	35.2	1,952	Human	Canada
*S. agalactiae* H36B	Ib	ST-6	NZ_AAJS00000000	Scaffolds	2,200,617	35.6	2,376	Human	USA
*S. agalactiae* ATCC 13813	II	ST-337	NZ_AEQQ00000000	Scaffolds	2,114,958	35.2	2,211	Bovine	UK
*S. agalactiae* FSL S3-026	III	ST-67	NZ_AEXT00000000	Scaffolds	2,455,848	36.1	2,334	Bovine	USA

### Identification of gene clusters

**Figure 1:**
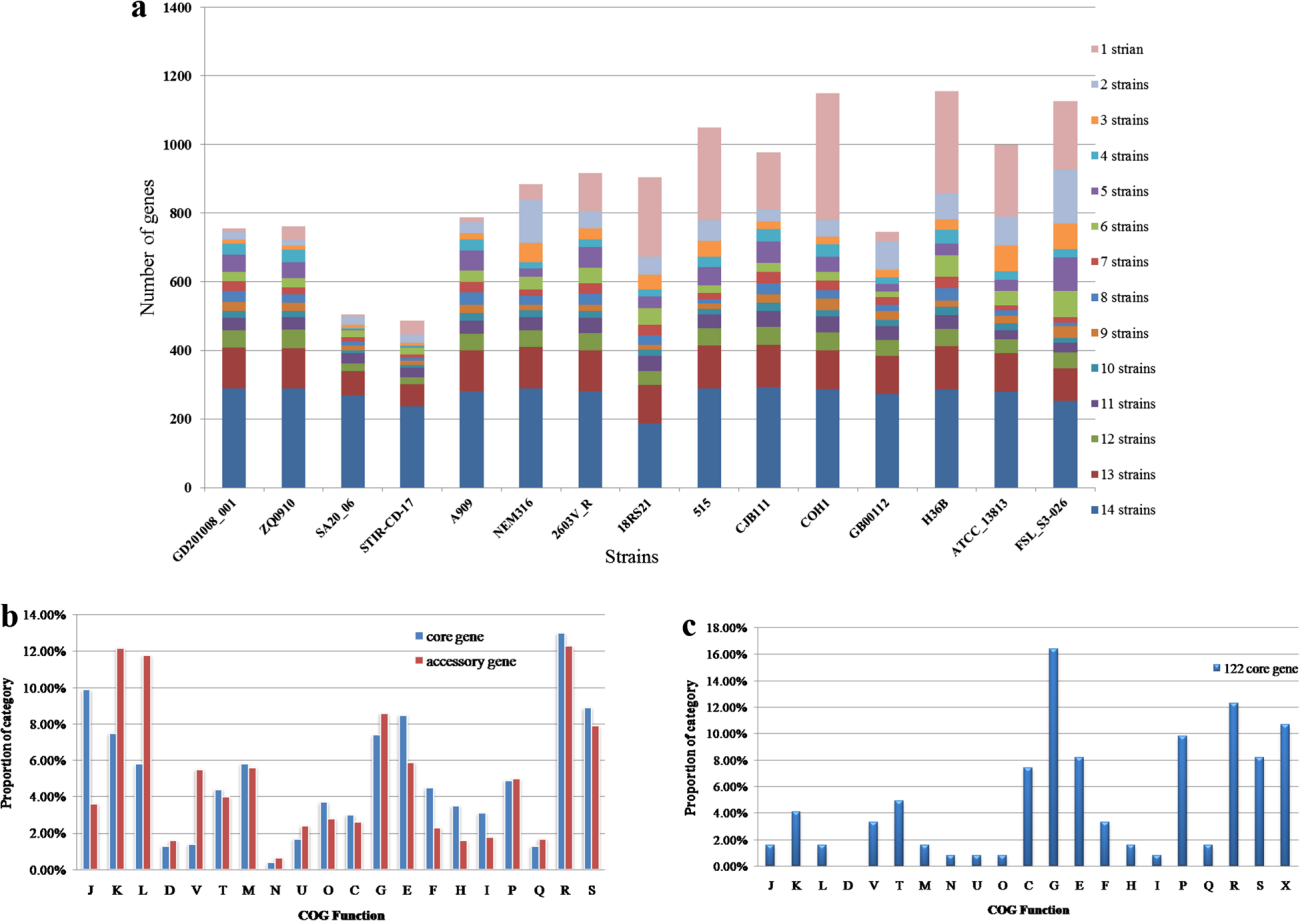
**The distribution of accessory gene number and genes functional classification of 15 *Streptococcus agalactiae *strains. (a)** Number of conserved accessory genes in each strain. All noncore genes in each strain were classified into different levels of conservation according to the number of strains. Different conservations are presented by various colors. **(b)** Comparison of COG functional categories between core and accessory genes. The Y-axis indicates the percentage of genes in a particular functional category relative to the genes of all COG categories. Only assigned COG functional genes were taken into account. **(c)** COG functional categories of 122 core genes. From the analysis of the core genome, 122 core genes have been deleted or suffered loss of function in the ST260-553 fish strains. The Y-axis indicates the percentage of genes in a particular functional category relative to the genes of all COG categories. Unclassified COG functional genes were taken into account.

The observed pan-genome shared by the 15 strains consisted of 4,730, genes including 1,202 core genes, 1,388 dispensable genes and 2,040 unique genes. The core genes accounted for 54.7% of CDSs of these strains. Accessory genes, including dispensable genes and unique genes, accounted for 10.4% of the total CDSs (32, 962 genes) from these 15 genomes; however, the distribution of accessory gene number in each strain varied considerably (Figure 1a). A COG functional classification for core and accessary genes was also performed. Only assigned COG functional genes were taken into account. Figure 1b shows that accessory genes were most likely to be assigned to COG categories K (Transcription), L (Replication, recombination and repair), V (Defense mechanisms), whereas core genes were more often associated with categories J (translation, ribosomal structure and biogenesis). However, 66% of the accessory genes were not assigned a COG function, reflecting novel gene clusters and the limitations of COG classification.

### The comparative analysis of genome elements

#### CRISPRs

CRISPRs (clustered regularly interspaced short palindromic repeats) are a family of DNA repeats that function like an adaptive immune response system, and are found in ~40% of bacteria. This system provides acquired immunity to exogenous DNA from viruses and plasmids, representing a barrier to attack or genetic transformation. According to Lopez-Sanchez et al. [14], there are two CRISPR/Cas systems in the genome of *S. agalactiae*. Type 1-C CRISPR2 is present in a few strains and type 2-A CRISPR1 is ubiquitous. BLAST results showed that the CRISPR2 components are missing or partially deleted in these 15 genome sequences. We then analyzed CRISPR1 sequences among the 15 *S. agalactiae* strains using the CRISPRs web server (http://crispr.u-psud.fr/) [15] and modified the results according Lopez-Sanchez et al.’s findings [14]. The Honduras fish isolates STIR-CD-17 and SA20-06 did not contain any CRISPR sequences. As in the other strains, GD201008-001 carried an acknowledged conserved CRISPR/cas locus of subtype II-A, with four cas genes [14]: cas9 (A964-0899, annotated as csn1 in the genome of GD201008-001), cas1 (A964-0900), cas2 (A964-0901) and csn2 (A964-0902, annotated as a hypothetical protein). Downstream of csn2, there was a 564 bp CRISPR region of GD201008-001 comprising a 36 bp direct repeat and eight spacers. The CRISPR details of 15 strains are listed in Additional file 1: Table S1.

**Figure 2:**
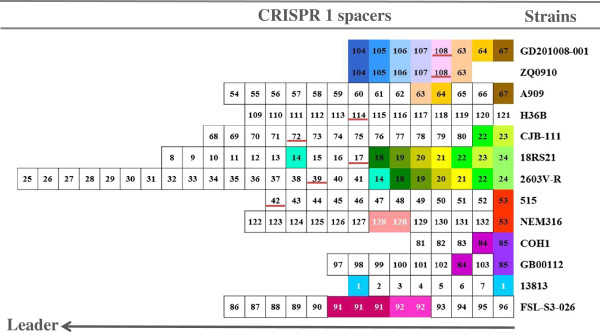
**Diversity of the CRISPR1 locus in 13 *Streptococcus agalactiae *strains.** Spacers were identified by the CRISPRtionary program, attributing a number to each spacer [19]. Repeats are not shown and spacers are represented in arbitrarily colored boxes. Strains with similar spacer arrays are arranged together and their names are given on the left. Single spacers appear in white boxes framed in black; identical spacers shared by different strains are shown with a same color background with black numbers; homologous spacers in the same strain are represented using the same color background and white numbers. The special spacers are underlined in red.

CRISPR spacers correspond to prior episodes of phage and plasmid exposure, and are inserted at the leader end of the CRISPR sequence, while the leader-distal end spacers are more conserved [16,17]. The sequence and position of spacers in the array could be used for limited ecological and epidemiological studies [18]. We identified 132 different spacer profiles from the 13 *S. agalactiae* strains with CRISPR1 sequences and grouped the strains according to the leader-distal spacers that corresponded to the most anciently inserted spacers [14]. To some extent, this allowed us to decipher phylogenetic relationships between strains. Figure 2 shows that two Chinese piscine strains, GD201008-001 and ZQ0910, and human isolate A909 share three identical spacers (spacer 63, 64, 67) in the leader-distal end. Human strains 18RS21 and 2603 V/R share six homologous pacers (spacer 18, 19, 20, 21, 22 and 24). Lopez-Sanchez et al. showed that the variability of CRISPR1 sequences could reflect the population structure and dissemination of *S. agalactiae* isolates [14]. Thus the strains sharing the same spacers were probably invaded by the same phage or plasmid, or lived in the same environment.

To identify the phages potentially targeted by CRISPR1 spacers, we searched for similar sequences in the NCBI nr database. A large number (71) of these 132 different spacers (54%) were found that perfectly or imperfectly matched known integrative and conjugative elements (ICE), or prophages in other streptococcal genomes, especially in *S. pyogenes* (9.8%). Spacers from GD201008-001, ZQ0910 and human strains 2603 V/R, 515, 18RS21, CJB111, H36B, respectively, matched different regions of the *S. agalactiae* lytic phage JX01 genome (Accession number: JX409895). Lytic phage JX01, which specifically infects *S. agalactiae*, was isolated from the milk of mastitis-affected cattle in China by our laboratory [20]. *S. agalactiae* strains GD201008-001 and 2603 V/R are resistant when challenged by *S. agalactiae* phage JX01, while A909 and ATCC 13813, with no spacers matching phage JX01 in our study, is also resistant to JX01 [20]. The CRISPR/cas system appears to be active against phages; however, there remain many factors influencing the host range of phages.

### Prophages

**Figure 3:**
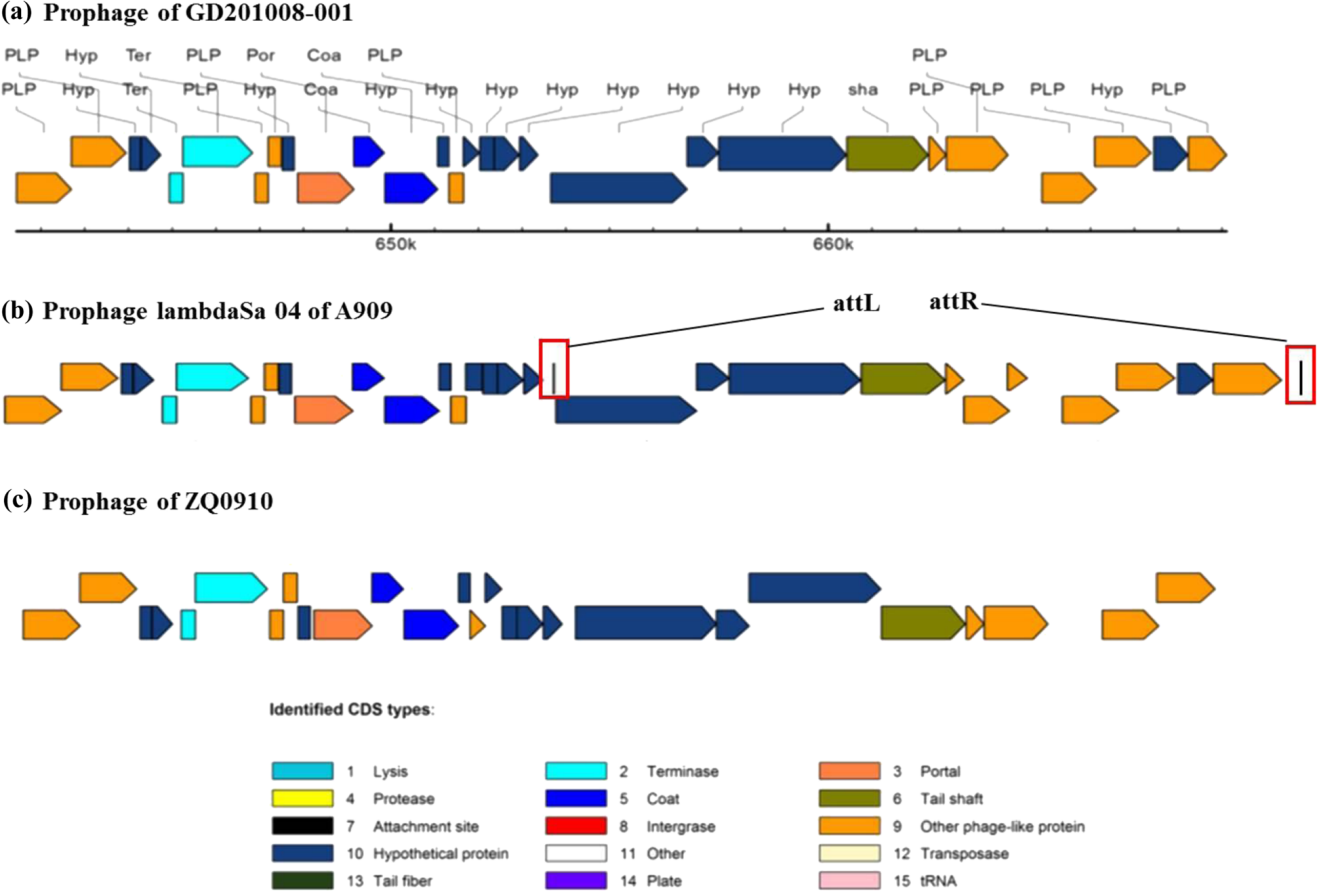
**Comparison of the CDs of the Prophage derived from *Streptococcus agalactiae *GD201008-001 (a), A909 (b) and ZQ0910 (c).** The detailed prophage views of three strains were produced using the online software PHAST (http://phast.wishartlab.com/index.html). Different colors represent various phage elements. The putative internal attL site and attR site of A909 prophage are labeled in white boxes framed in red.

With the exception of fish isolate SA20-06 and human isolates NEM316 and COH1, twelve strains contained prophage sequences. Their features are listed in Additional file 1: Table S2. GD201008-001 contained a 28 kb prophage encoding 29 CDSs. Subsequent global nucleotide alignment revealed that the prophage of GD20108-001 had high sequence identity (99%) with the Chinese tilapia isolate ZQ0910 and the *S. agalactiae* A909 LambdaSa04 prophage, while the location of prophages in these three strains was also similar (Figure 3). One difference among these three prophages is that A909 contains a putative internal *attL* site and an *attR* site at the end of the prophage. Therefore, it is possible that GD201008-001, ZQ0910 and A909 exchanged the phage within a shared human or fish environment. Other available fish *S. agalactiae* genomes do not appear share any common prophage genes with human isolates; therefore, it is also possible that Chinese piscine strains GD201008-001 and ZQ0910 were derived from a human source.

### Virulence factors in piscine strains

As pathogenic strains, A909 and piscine strains (GD201008-001, ZQ0910, SA20-06 and STRI-CD-17) encode most of the known *S. agalactiae* virulence factors (Additional file 1: Table S3). They contain several genes encoding adherence related proteins, such as fibrinogen-binding proteins (fbsA, fbsB, pavA), pilus islands (PI), immunogenic bacterial adhesin (BibA) and invasion-associated protein (iagA).

There are three pilus island variants (PI-1, 2a, 2b) in GBS that are associated with enhanced bacteria phagocyte resistance and systemic virulence [21]. Four piscine strains carried the same pilus island type 2b (PI-2b), but no PI-1 or PI-2a, while human strain A909 has PI-1 and PI-2b. Meanwhile, the PCR detection of the backbone of pilus islands PI-1, PI-2 and PI-2b, as described [22], were performed among 21 fish strains from three fisheries in China in our study. The results (Additional file 1: Table S4) were confirmed the former conclusion that at least one of the three pilus islands were present in each strain of *S. aglactiae*[22]. The fish isolates in our study carried either PI-2b (52%) or PI-1 plus PI-2b type (48%), but no PI-2a. In human infection, there was a large proportion of GBS from adults that carry both PI-1 and PI-2a, and a greater number of strains in infants bearing both PI-1 and PI-2b [22]. This suggests that *S. agalactiae* probably changed its pili type under host immunological pressure to expand its capacity to infect different hosts. This hypothesis should be tested by examining the pilus type among hundreds of piscine isolates.

We also analyzed single nucleotide polymorphisms (SNPs) in genes surrounding the PI-1 locus. Ten genes from the upstream and the downstream regions, respectively, of the A909 PI-1 locus were chosen to compare with the GD201008-001 genome. We identified 3 polymorphisms, comprising 1 non synonymous (ns) SNPs, 1 synonymous (s) SNPs, and 1 deletions (see Additional file 1: Table S5). The result showed that the absence of a PI-I locus was not the result of a simple deletion event, and may have occurred by recombination with a more distantly related genome.

Other streptococcal virulence proteins, such as hyaluronidase, enolase, surface immunogenic protein (sip), pneumococcal surface antigen A (psaA), C3-degrading protease, Trigger factor and CAMP factor were identified in these five strains. Two immunoreactive antigens, the Alpha C protein (ACP), known to mediate GBS invasion of human cervical epithelial cells [23], and the beta C protein (BCP), which binds human IgA to evade human immune responses [24], were only found in human strain A909 and Chinese piscine isolates GD201008-001, ZQ0910, A909, GD201008-001 and ZQ0910. These strains are hemolytic and contain the complete hemolysin operon (*cylX, cylD, cylG,acpC, cylZ, cylA, cylB, cylE, cylF, cylI, cylJ, cylK*) in their genome, while SA20-06 and STRI-CD-17, which are non-hemolytic [5,25], have an incomplete operon containing only the *cylA*, *cylB* and *cylE* genes. This suggests that the final secretion of hemolysin depends on the complete hemolysin operon, although cylE has been proposed to be the structural gene hemolysin of *S. agalactiae*[26]. In addition, all the piscine *S. agalactiae* genomes lack the genes *lmb* and *scpB*, which encode Laminin-binding protein and C5a peptidase, respectively. To date, no *lmb* or *scpB* genes have been published in the NCBI nr database that are derived from piscine *S. agalactiae* strains. This phenomena should be studied further in more piscine strains. Thus, *S. agalactiae* has retained indispensable genes for survival and reproduction and has lost some nonessential genes during adaptation to various niches in different hosts.

### Core and pan-genome analysis of *S. agalactiae*

#### *S. agalactiae* core genome

**Figure 4:**
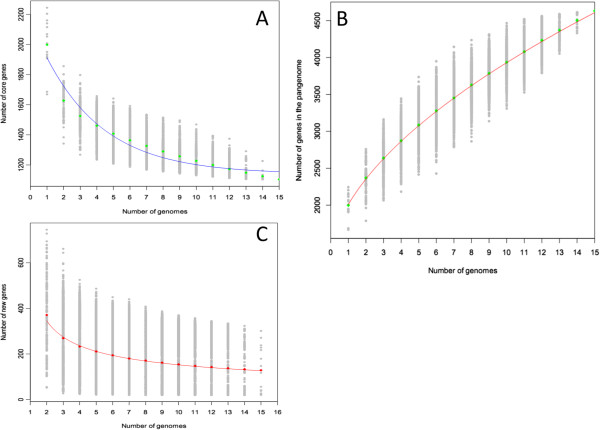
**Core and pan-genome calculations for 15 *S. agalactiae *strains. (A) ***S. agalactiae* core genome. Each point represents the number of conserved genes between genomes. They are plotted as functions of strain number (x). For each x, circles are the 15!/[(x-1)!(15-x)] values from the different strain combinations. Squares are the averages of such values. The blue line represents the least-squares fit of the function C(x) = Ac x ^–tc^ + yc. The best fit was obtained with correlation r^2^ = 0.960 for Ac = 1021士80, tc = 0.28, yc = 1140士8. **(B) ***S. agalactiae* pan-genome. Numbers of genes are calculated for all possible combinations and plotted as a function of strain numbers (x). The red line demonstrates the exponential model based on the mean value of pan genes. The deduced pan-genome size P(x) = As*x^(ts) + ys. The best fit was obtained with correlation r^2^ = 0.999 for As = 726士2, ts = 0.562, ys = 1284士7. **(C)** Number of new predicted gene clusters identified by the sequencing additional genomes. The curve is fitted to the function S(x) = As*x^(ts)-As*(x-1)^(ts).

The number of core genes found after the sequential addition of each new genome sequence was extrapolated by fitting an exponential decaying function to the data [27]. The number of shared genes initially decreased with addition of each new sequence. Figure 4A demonstrates that the core genome size of 15 *S. agalactiae* strains is 1,202 genes and is likely to decrease substantially with the sequencing of additional genomes. This result was different from the Tettelin et al.’s study [27], which showed that the core genome of eight human strains comprised 1,806 genes and remained constant even when more genomes were added. The differences in the absolute numbers reflect the differences in methodology used to define orthology [27], and the use of draft genome sequence data. To insure the robustness of our method, we also analyzed the core genes of these eight human strains. The result was 1470 core genes, extremely closed to the results of Lefebure‘s analysis of the core genes of the eight human strains (1472 core genes) [28].

The genomes size of the two Latin American fish strains, SA20-06 (ST-553) and STIR-CD-17 (ST-260) was 1.8 Mbp, obviously smaller than the other 13 *S. agalactiae* strains. Would these two strains cause decrease in the core genome size of *S. agalactiae*? We analyzed the core genome from 13 strains after removing these two strains. There were 1324 core genes among the 13 *S. agalactiae* strains, much higher than 1202 core genes from 15 strains. This 122 genes appear to have been deleted in the ST260-553 strains genomes. The two Latin American fish strains’ genomes decreased the core genome size of *S. agalactiae* in our study. From Rosinski-Chupin’s study [29], specialized fish strains ST260-261-553 are characterized by a strong reductive evolution, which led to the relaxation of the negative selection on genes that became dispensable, allowing the accumulation of larger deletions. These 122 core genes were also subjected to COG functional classification (Figure 1c). Unclassified COG functional genes were taken into account. Among these 122 core genes, ST260-553 fish strains were most likely to delete genes in COG categories G (Carbohydrate transport and metabolism) and R (General function prediction only), while the core genes among 15 strains were more often associated with R (General function prediction only) and J (translation, ribosomal structure and biogenesis) (Figure 1b). These data suggested that in the evolution of the *S. agalactiae* strains specialized to fish, some proteins associated with carbohydrate transport and metabolism functions were deleted linking to host adaptation.

#### *S. agalactiae* pan-genome

For the pan-genome study, the number of new genes (unique genes) was calculated every time a new genome was incorporated. The mean values of new genes were used to perform the extrapolation. Similar to the core genes, the plot of new genes fitted well to a decaying function, and the extrapolated curve did not stabilize on a non zero asymptotic value before the genome number reached 15, as shown in Figure 4C. It meant that the number of new genes in *S. agalactiae* was not characterized by those 15 genomes and would tend to a non-zero asymptotic value when more genomes are added. Figure 4B shows that our results agree with Tettelin et al.’s: *S. agalactiae* possesses an open pan-genome whose size increases with the addition of new sequenced strains.

### Phylogenetic relationships among *S. agalactiae* strains from different sources

**Figure 5:**
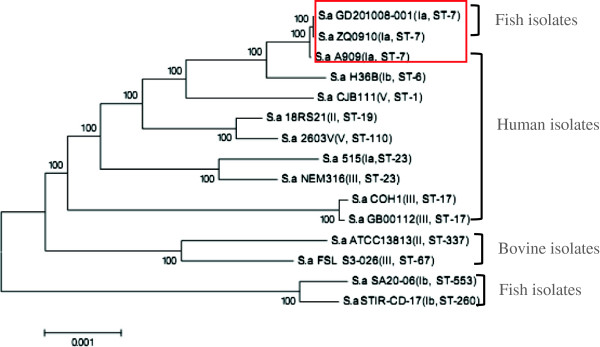
**Phylogenetic tree showing the relationship among 15 *S. agalactiae *strains based on 1114 orthologous gene clusters.** A neighbor-Joining (NJ) phylogenetic tree based on the 1,114 single copy orthology clusters of the 15 strains.

**Figure 6:**
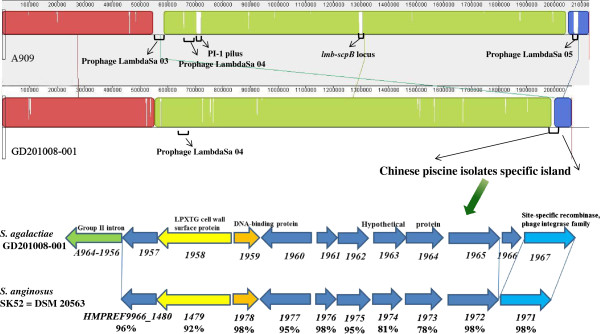
**Whole genome alignment between *S. agalactiae *GD201008-001 and A909 and Chinese piscine isolates specific island.** The genomes of GD201008-001 and A909 were compared with each other using progressive MAUVE with default parameters, and the colinearity of the genomes is shown. Human strain A909 has two additional prophage sequences (prophage LambdaSa 03 (548,964 bp-585,049 bp), prophage LambdaSa 05 (2,069,932 bp-2,086,164 bp)), PI-1 type pilus(711,448 bp -720,303 bp) ) and *lmb-scpB* locus (1,293,687 bp -1,303,948 bp), but lacked the Chinese piscine *S. agalactiae* specific genes island (1,992,339 bp -2,002,579 bp in GD201008-001), which only matched the region of *Streptococcus anginosus* SK52 = DSM 20563 with high level amino acid identity (78-98%).

To investigate the phylogenetic relationships among these fifteen strains, we used the neighbor-joining (NJ) method to construct a phylogenetic tree (Figure 5) using the 1,114 single copy orthology clusters of the fifteen strains. As expected, identical host origin strains showed close evolutionary relationships, such as fish sources (STIR-CD-17 and SA20-06), bovine sources (ATCC 13813 and FSL S3-026) and human sources (18RS21 and 2603 V/R, 515 and NEM316, COH1 and GB00112). Serotype III, ST 17 human strains COH1 and GB0012, serotype Ib piscine strains STIR-CD-17 and SA20-06, respectively belonged to the same evolutionary clade. Human strain A909 and piscine Chinese isolates GD201008-001 and ZQ0910, which belong to the same serotype and ST (Ia, ST7), were also grouped into one evolutionary branch. This indicates that Chinese fish strains GD201008-001 and ZQ0910 were probably derived from the same recent common ancestor as human strain A909. We then evaluated GD201008-001 and A909 at the genomic level (Figure 6). The whole genome alignment indicated that A909 has additional two prophage sequences (prophage LambdaSa 03, 05), a PI-1 pilus locus and *lmb-scpB* locus, but lacked the Chinese piscine *S. agalactiae* specific genes island. In general, the position of four piscine *S. agalactiae* strains (GD201008-001, ZQ0910, STIR-CD-17 and SA20-06) on the phylogenetic tree indicated that Chinese piscine *S. agalactiae* strains were more closed related to human strains than to Latin American fish strains.

### Specific genes among piscine strains

Compared to the human GBS genomes, 131 orthologs were identified as specific to the piscine GBS genomes (GD201008-001, ZQ0910, SA20-06, STIR-CD-17) and 63 (48%) genes were annotated as encoding “hypothetical proteins”. Among these 131 genes, 98 genes (75%) were found only in a single strain, 10 belonging to GD201008-001, 41 belonging to ZQ0910, eight belonging to SA20-06 and 39 belonging to STIR-CD-17. There were no genes specifically shared by these four piscine strains. Many of the piscine strain-specific genes (66%) were partial, caused by base mutation or insertion elements. A strain-specific 10 kb gene island was found in Chinese isolates GD201008-001 and ZQ0910 [8], which contains one LPXTG cell wall surface protein, one DNA binding protein, one phage integrase, and eight hypothetical proteins. This 10 kb locus shows significant sequence similarity and identical gene order to the genome of *Streptococcus anginosus* SK52/DSM 20563 (Figure 6), which agreed with the results of Rosinski-Chupin et al. [29]. Although some of the genes within this 10 kb island showed close sequence similarity to genes from other *S. agalactiae* strains, as well as tilapia strain STIR-CD-21 and human strain GB00206, *Streptococcus anginosus* SK52/DSM 20563 was the only species that matched the entire region with high amino acid identity (78-98%). *Streptococcus anginosus* is part of the human bacteria flora, but can cause diseases, including brain and liver abscesses, under certain circumstances. This 10 kb locus in *S. agalactiae* may have been acquired from *Streptococcus anginosus* and may contribute to the virulence of piscine strains.

## Conclusions

In summary, comparative genomic analysis among 15 *S. agalactiae* strains of different origins showed that the open pan-genome of these *S. agalactiae* comprises 4,730 genes including 1,202 core genome genes, 1388 dispensable genes and 2040 strain-specific genes. The Latin American fish-specific strains SA20-06 and STIR-CD-17, belonging to ST260-553, decrease the core genome size of *S. agalactiae* and are quite distinct from Chinese fish isolates GD201008-001 and ZQ0910 at the genome level. Except for core genes, no genes are specifically shared by these four piscine strains.

The Chinese piscine *S. agalactiae* GD201008-001 and ZQ0910, belonging to ST 7, are very similar to their human counter-part, A909. They are highly similar in the context of CRISPR, prophage, and virulence-associated genes, and share an extremely short evolutionary relationship in the phylogenetic tree. There is no evidence showing that humans could be infected by piscine *S. agalactiae* through wounds or consuming fish meat. However, attention must be paid to its potential threat to public health security because these ST7 fish strains are not distinguished from their human counterparts. In addition to their genomic similarities, a transcriptome study of Chinese piscine *S. agalactiae* will be performed to detect and compare gene expression with human ST 7 strains in our future research.

## Methods

### Bacterial strains

The genome sequence of piscine strain GD201008-001 has been deposited in the GenBank database under the accession numbers CP003810. Fourteen *S. agalactiae* strains genomes representing three different host tropisms were downloaded from GenBank and are listed in Table 1.

### Genome element prediction

CRISPRs finder (http://crispr.u-psud.fr/) [15], was used to identify clustered regularly interspaced short palindromic repeats (CRISPRs). PHAST (http://phast.wishartlab.com/index.html) [30] was used to identify prophage sequences. Amino acid sequences of the CDSs of four piscine strains and human strain A909 were searched against the Virulence Factor of Pathogenic Bacteria database (VFDB, http://www.mgc.ac.cn/VFs/main.htm) using BLASTp. We used an E value cut-off of 1e-5 and retained the single best hit.

### Orthology reconstruction

OrthoMCL version 2.0 [31] was used to delineate orthologous protein sequences among the fifteen *S. agalactiae* genomes. The first step in the OrthoMCL procedure was to perform an all-against-all BLASTp search within and between all genome pairs. We grouped the genes into clusters that share 50% sequence identity over 50% of the protein gene length. A *P*-value cut-off of 1e-5 was chosen for putative orthologs or paralogs. Putative orthologous and paralogous relationships were then converted into a graph in which the nodes represent protein sequences, and the weighted edges represent their relationships. Based on pre-computed sequence similarity information, a Markov cluster (MCL) algorithm was used to assign proteins into families [32].

### Distribution of pilus-like structure genes among Chinese piscine isolates

PCR analysis was performed to detect the backbone of pilus island PI-1, PI-2 and PI-2b, as described previously [22], using Chinese piscine isolates’ genomes as templates.

### Core and pan-genome analysis

Tables of homologous clusters from OrthoMCL were compiled to identify shared and unique genes. Fifteen strains with complete genome sequences were simulated in all possible combinations. The sizes of the core genome and the novel gene set were calculated for each combination and then extrapolated using several functions to find a best fit from the mean number at each sampling point [27,33].

### Phylogenetic tree building

A phylogenetic tree of *S. agalactiae* strains was constructed based on homologous clusters from OrthoMCL. The 1,114 single-copy core genes were used to perform, multiple sequence alignment using MUSCLE v3.7 [34]. The alignments of these genes were concatenated into a single sequence alignment with a length of 102,9476 bp, and a neighbor-joining tree was reconstructed using MEGA (Version 5.05).

## Competing interests

The authors declare that they have no competing interests.

## Authors’ contributions

GJL and CPL carried conceived the study and wrote the article; WZ and GJL analyzed the sequence data. All authors read and approved the final manuscript.

## Supplementary Material

Additional file 1: Table S1 CRISPRs found in fifteen *S.agalactiae* strains. **Table S2**. Predicted prophage regions for each strain. **Table S3**. Virulence-related genes in piscine strians and A909. **Table S4**. Pilus type of *Streptococcus agalactiae* fish isolates in China. **Table S5**. SNPs of PI-1 locus between A909 and GD201008-001. Click here for file
