# Supplementary material for: Comparative genomics analysis of Streptococcus agalactiae reveals that isolates from cultured tilapia in China are closely related to the human strain A909
Source: BMC Genomics. 2013 Nov 11;14:775. doi: 10.1186/1471-2164-14-775 (PMC3831827; doi:10.1186/1471-2164-14-775)
Supplement: Additional file 1: Table S1 — CRISPRs found in fifteen S.agalactiae strains. Table S2. Predicted prophage regions for each strain. Table S3. Virulence-related genes in piscine strians and A909. Table S4. Pilus type of Streptococcus agalactiae fish isolates in China. Table S5. SNPs of PI-1 locus between A909 and GD201008-001. [file 1471-2164-14-775-S1.doc]

**Additional file**

**Table S1** **CRISPRs found in fifteen *S.agalactiae*** strains

| **Strain** | **CRISPR Number** | **CRISPR Length(bp)** | **Spacer Number** | **Special Spacer*** | **BLAST Result** |
| --- | --- | --- | --- | --- | --- |
| **GD201008-001** | 1 | 564 | 8 | CAATTGATTGCCGTTAAAACCGATAGAGGA | 100% identity of Streptococcus phage JX01 minor structural protein (orf 50) gene |
| **SA20-06** | 0 | - | - | - | - |
| **ZQ0910** | 1 | 431 | 6 | CAATTGATTGCCGTTAAAACCGATAGAGGA | 100% identity of Streptococcus phage JX01 minor structural protein (orf 50) gene |
| **STIR-CD-17** | 0 | - | - | - | - |
| [**A909**](http://www.ncbi.nlm.nih.gov/genome/186?project_id=57935) | 1 | 959 | 14 | - | - |
| [**NEM316**](http://www.ncbi.nlm.nih.gov/genome/186?project_id=61585) | 1 | 893 | 13 | - | - |
| [**2603V/R**](http://www.ncbi.nlm.nih.gov/genome/186?project_id=57943) | 1 | 1619 | 24 | GCGATGATGGTAAGTCATCATGGACAGCGT | 100% identity of Streptococcus phage JX01 minor structural protein (orf 51) gene |
| [**18RS21**](http://www.ncbi.nlm.nih.gov/genome/186?project_id=54309) | 1 | 1157 | 17 | ACGGTGTTGCACACTCTATCACTTATAAAAA | 100% identity of Streptococcus phage JX01 minor structural protein (orf 52) gene |
| [**515**](http://www.ncbi.nlm.nih.gov/genome/186?project_id=54311) | 1 | 827 | 12 | TGTACAAATACAGGATACGCAAGGGATATA | 96% identity of Streptococcus phage JX01 lysin (orf 44) gene; 100% identity of S.agalactiae bacteriophage NCTC11261 lysin PlyGBS gene |
| **CJB111** | 1 | 1156 | 17 | TATCAGTCCACTTATAATCAAGATAGTTTG | 100% identity of Streptococcus phage JX01 minor structural protein (orf 51) gene |
| **COH1** | 1 | 336 | 5 | - | - |
| **GB00112** | 1 | 629 | 9 | - | - |
| **H36B** | 1 | 892 | 13 | CAATTGCATTAACTTTTGCAATGCTTTTAT | 100% identity of Streptococcus phage JX01 Putative uncharacterized protein  (orf 49) gene |
| [**ATCC 13813**](http://www.ncbi.nlm.nih.gov/genome/186?project_id=68679) | 1 | 563 | 8 | - | - |
| **FSL S3-026** | 1 | 959 | 14 | - | - |

* : Special Spacer**:** the sequence of those spacers matched certain region in the *S. agalactiae* lytic phage genome

-: no matched result.

**Table S2. P**redicted prophage regions for each strain

| **Strain** | **REGION** | **LENGTH (kb)** | **COMPLETENESS(score)** | **SPECIFIC_KEYWORD** | **REGION_POSITION** | **MOST_COMMON_PHAGE_NAME** | **GC%** |
| --- | --- | --- | --- | --- | --- | --- | --- |
| **GD201008-001** | 1 | 28.0* | Intact (90) | terminase, portal, capsid, head, lysis, recombinase | 641012-669106 | PHAGE_Strept_PH10 | 42.9 |
| **SA20-06** | 0 | - | - | - | - | - | - |
| **ZQ0910** | 2 | 28.0* | Intact (90) | recombinase, lysis, head, capsid, portal, terminase | 1395269-1423365 | PHAGE_Strept_PH10 | 42.8 |
|  |  | 33.9 | Questionable (80) | transposase, integrase | 1438400-1472366 | Prophage Escherichia coli str. K-12 substr. MG1655 | 33.6 |
| **STIR-CD-17** | 1 | 34.1 | Incomplete (50) | integrase, recombinase | 45975-80098 | Cafeteria roenbergensis virus BV-PW1 |  |
| **A909** | 3 | 45.2 | Questionable (80) | lysin, integrase, terminase, plate, tail | 540785-586079 | Streptococcus_pyogenes_phage_315_3 | 36.6 |
|  |  | 31.2* | Intact (100) | terminase, portal, capsid, head, lysis, recombinase | 675770-707032 | PHAGE_Strept_PH10 | 41.4 |
|  |  | 34.5 | Incomplete (30) | integrase | 2051604-2086164 | Lactococcus_phage_bIL311 | 34.6 |
| [**NEM316**](http://www.ncbi.nlm.nih.gov/genome/186?project_id=61585) | 0 | - | - | - | - | - | - |
| **2603V/R** | 3 | 66.7 | Intact (140) | transposase, integrase, terminase, plate, tail, lysin | 550498-617294 | Streptococcus_pyogenes_phage_315_3 | 35.3 |
|  |  | 59.5 | Questionable (90) | lysin, capsid, head, portal, terminase, integrase | 1816883-1876441 | PHAGE_Strept_PH10 | 38.5 |
|  |  | 33.5 | Incomplete (40) | integrase | 1944852-1978401 | Lactococcus_phage_bIL309 | 35.1 |
| **18RS21** | 2 | 21.0 | Incomplete (60) | protease, tail, capsid, terminase | 945178-966182 | Streptococcus_phage_Cp_1 | 37.2 |
|  |  | 37.0 | Incomplete (40) | protease, integrase | 1424044-1461066 | Streptococcus_phage_2972 | 34.8 |
| **515** | 2 | 41.8 | Intact (130) | terminase, capsid, tail, lysin, integrase, transposase | 625025-666919 | Streptococcus_phage_Cp_1 | 35.6 |
|  |  | 27.3 | Incomplete (30) | integrase | 1423093-1450471 | Temperate_phage_phiNIH1_1 | 36.4 |
| **CJB111** | 2 | 29.7 | Questionable (80) | terminase, portal, capsid, head, lysis, recombinase | 785542-815265 | PHAGE_Strept_PH10 | 40.6 |
|  |  | 15.5 | Incomplete (30) | transposase | 2066525-2082080 | Ictalurid_herpesvirus_1 | 36.9 |
| **COH1** | 0 | - | - | - | - | - | - |
| **GB00112** | 2 | 15.6 | Incomplete (20) | integrase | 247565-263221 | Lactococcus_phage_bIL310 | 36.2 |
|  |  | 47.0 | Questionable (90) | lysin, integrase, terminase, tail | 1401637-1448695 | Streptococcus_pyogenes_phage_315_6 | 36 |
| **H36B** | 2 | 53.9 | Questionable (80) | lysin, integrase, terminase, tail | 327210-381206 | Streptococcus_pyogenes_phage_315_6 | 36.2 |
|  |  | 32.2 | Incomplete (40) | integrase, protease | 1047144-1079439 | Acanthamoeba polyphaga mimivirus | 34.4 |
| **ATCC 13813** | 4 | 15.8 | Incomplete (20) | integrase | 55181-71061 | Streptococcus_phage_5093 | 36.5 |
|  |  | 38.6 | Intact (100) | recombinase, terminase, capsid, tail | 298366-337002 | Streptococcus_phage_Cp_1 | 37.1 |
|  |  | 18.9 | Incompete (20) | integrase | 1838420-1857327 | Bacillus phage B4 | 32.9 |
|  |  | 16.1 | Questionable (90) | transposase | 2097373-2113566 | Lactobacillus_phage_phiAT3 | 37.1 |
| **FSL S3-026** | 3 | 24.1 | Incomplete (60) | integrase, transposase | 260914-285026 | Streptococcus_pyogenes_phage_315_6 | 36.9 |
|  |  | 60.8 | Intact (120) | terminase, portal, capsid, head, lysis, lysin, recombinase, transposase | 781883-842689 | Streptococcus_phage_PH10 | 40.9 |
|  |  | 27.5 | Intact (110) | recombinase, lysin, lysis, head, capsid, portal, terminase | 1247728-1275284 | Streptococcus_phage_PH10 | 43.9 |

*: share 99 % identical sequence

-: no matched result.

Table S3. Virulence-related genes in piscine strians and A909

| **Virulence factors** | **Related genes** | **A909** | **GD201008-001** | **ZQ0910** | **SA20-06** | **STIR-CD-17** |
| --- | --- | --- | --- | --- | --- | --- |
| ***Adhesin*** | | | | | | |
| **fibrinogen-binding protein** | pavA | SAK_1277 | A964_1163 | WY5_04360 | SaSA20_1009 | M3M_03075 |
|  | fbsA | SAK_1142 | A964_1028 | WY5_05060 | SaSA20_0877 | M3M_07935 |
|  | fbsB | SAK_0955 | A964_0836 | WY5_06055 | SaSA20_0708 | — |
| **Laminin-binding protein** | lmb | SAK_1319 | — | — | — | — |
| **Pilus island 1** | PI-1 backbone protein | SAK_0776 | — | — | — | — |
|  | PI-1 ancillary protein 2 | SAK_0777 | — | — | — | — |
|  | sortase family protein | SAK_0778 | — | — | — | — |
|  | sortase family protein | SAK_0779 | — | — | — | — |
|  | PI-1 ancillary protein 1 | SAK_0780 | — | — | — | — |
| **Pilus island 2a** | PI-2a ancillary protein 2 | — | — | — | — | — |
|  | sortase family protein | SAK_1439 | A964_1320 | WY5_03585 | SaSA20_1146 | — |
|  | sortase family protein | — | — | — | — | — |
|  | PI-2a backbone protein | — | — | — | SaSA20_1147 | — |
|  | PI-2a ancillary protein 1 | — | — | — | — | — |
| **Pilus island 2b** | PI-2b ancillary protein 1 | — | A964_1322 | WY5_03575 | SaSA20_1149 | M3M_06299 |
|  | PI-2b backbone protein | SAK_1440 | A964_1321 | WY5_03580 | SaSA20_1148 | M3M_06294 |
|  | sortase family protein | SAK_1439 | A964_1320 | WY5_03585 | SaSA20_1146 | — |
|  | PI-2b ancillary protein 2 | SAK_1438 | A964_1319 | WY5_03590 | SaSA20_1145 | M3M_06274 |
|  | sortase family protein | SAK_1437 | A964_1318 | WY5_03595 | SaSA20_1144 | M3M_06269 |
| **Immunogenic bacterial adhesin** | BibA | SAK_2002 | A964_1909 | WY5_00661 | SaSA20_1675 | M3M_09338 |
| **invasion-associated gene** | iagA | SAK_0835 | A964_0709 | WY5_06685 | SaSA20_0586 | M3M_00150 |
| ***Exoenzyme*** | | | | | |  |
| **Hyaluronidase** | hylB，hyaluronate lyase | SAK_1284 | A964_1170 | WY5_04325 | SaSA20_1016 | M3M_03035 |
| **Streptococcal enolase** | eno,phosphopyruvate hydratase | SAK_0713 | A964_0597 | WY5_07250 | SaSA20_0534 | M3M_00505 |
| ***Immunoreactive antigen*** | | | | | |  |
| **Alpha C protein** | bca | SAK_0517 | A964_0444 | WY5_08027 | — | — |
| **Beta C protein** | cba | SAK_0186 | A964_0140 | WY5_08891 | — | — |
| **Surface immunogenic protein** | sip | SAK_0065 | A964_0031 | WY5_10073 | SaSA20_0031 | M3M_05317 |
| ***Metal transport*** | | | | | |  |
| **Pneumococcal surface antigen A / Metal binding protein SloC** | psaA | SAK_1556 | A964_1440 | WY5_02975 | SaSA20_1259 | M3M_07054 |
| ***Protease*** | | | | | |  |
| **C3-degrading protease** | cppA | SAK_1738 | A964_1635 | WY5_02013 | SaSA20_1438 | M3M_02188 |
| **C5a peptidase** | scpA/scpB | SAK_1320 | — | — | — | — |
| **Serine protease** | htrA/degP，serine protease | SAK_2135 | A964_2021 | WY5_00085 | SaSA20_1771 | M3M_07349 |
| **Trigger factor** | tig/ropA | SAK_0155 | A964_0108 | WY5_08729 | SaSA20_0103 | M3M_04650 |
| ***Toxin*** | | | | | |  |
| **Beta-hemolysin/cyto-lysin** | cylX | SAK_0790 | A964_0658 | WY5_06935 | — | — |
|  | cylD | SAK_0791 | A964_0659 | WY5_06930 | — | — |
|  | cylG | SAK_0792 | A964_0660 | WY5_06925 | — | — |
|  | acpC | SAK_0793 | A964_0661 | WY5_06920 | — | — |
|  | cylZ | SAK_0794 | A964_0662 | WY5_06915 | — | — |
|  | cylA | SAK_0795 | A964_0663 | WY5_06910 | SaSA20_0555 | M3M_00355 |
|  | cylB | SAK_0796 | A964_0664 | WY5_06905 | SaSA20_0556 | M3M_00350 |
|  | cylE | SAK_0797 | A964_0665 | WY5_06900 | SaSA20_0557 | M3M_00345 |
|  | cylF | SAK_0798 | A964_0666 | WY5_06895 | — | — |
|  | cylI | SAK_0799 | A964_0667 | WY5_06890 | — | — |
|  | cylJ | SAK_0800 | A964_0668 | WY5_06885 | — | — |
|  | cylK | SAK_0801 | A964_0669 | WY5_06880 | — | — |
| **CAMP factor** | cfa/cfb | SAK_1983 | A964_1892 | WY5_00746 | SaSA20_1659 | M3M_09048 |

—: no matched result.

Table S4. Pilus type of *Streptococcus agalactiae* ﬁsh isolates in China

| **Isolate** | **Host** | **Origin** | **PI component(s)** |
| --- | --- | --- | --- |
| **ATCC 13813** | Cattle | UK | PI-2b |
| **NEM316** | human | France | PI-1 plus PI-2a |
| **2603V/R** | human | USA | PI-1 plus PI-2a |
| **A909** | Human | USA | PI-1 plus PI-2b |
| **GD201008-001** | tilapia | Guangdong-1 | PI-2b |
| **GD201008-002** | tilapia | Guangdong-1 | PI-2b |
| **GD201008-003** | tilapia | Guangdong-1 | PI-2b |
| **GD201008-004** | tilapia | Guangdong-1 | PI-2b |
| **GD201008-005** | tilapia | Guangdong-1 | PI-2b |
| **GD201008-006** | tilapia | Guangdong-1 | PI-2b |
| **GD201008-007** | tilapia | Guangdong-1 | PI-2b |
| **GD201008-008** | tilapia | Guangdong-2 | PI-1 plus PI-2b |
| **GD201008-009** | tilapia | Guangdong-2 | PI-1 plus PI-2b |
| **GD201008-010** | tilapia | Guangdong-2 | PI-1 plus PI-2b |
| **GD201008-011** | tilapia | Guangdong-2 | PI-1 plus PI-2b |
| **GD201008-012** | tilapia | Guangdong-2 | PI-1 plus PI-2b |
| **GD201008-013** | tilapia | Guangdong-2 | PI-1 plus PI-2b |
| **GD201008-014** | tilapia | Guangdong-2 | PI-1 plus PI-2b |
| **GD201008-015** | tilapia | Guangdong-3 | PI-2b |
| **GD201008-016** | tilapia | Guangdong-4 | PI-1 plus PI-2b |
| **GD201008-017** | tilapia | Guangdong-4 | PI-1 plus PI-2b |
| **GD201008-018** | tilapia | Guangdong-4 | PI-1 plus PI-2b |
| **GD201008-019** | tilapia | Guangdong-4 | PI-1 plus PI-2b |
| **GDMM** | tilapia | Guangdong-5 | PI-1 plus PI-2b |
| **SD201009** | tilapia | Shandong | PI-2b |
| **HN-2** | tilapia | Hainan | PI-2b |
| **HN-6** | tilapia | Hainan | PI-2b |
| **HN-7** | tilapia | Hainan | PI-2b |
| **HN-9** | tilapia | Hainan | PI-2b |

Table S5. SNPs of PI-1 locus between A909 and GD201008-001

| **ORF from A909** | **ORF from GD201008-001** | **SNP**  **locus** | **DNA polymorphism** | **amino acid polymorphism** | **SNP types** |
| --- | --- | --- | --- | --- | --- |
| SAK_0754 | A964_0637 | — |  |  |  |
| SAK_0756 | A964_0638 | — |  |  |  |
| SAK_0757 | A964_0639 | — |  |  |  |
| SAK_0758 | A964_0640 | — |  |  |  |
| SAK_0759 | A964_0641 | 1404 | GGT/GGC | G/G | synonymous |
| SAK_0760 | A964_0642 | — |  |  |  |
| SAK_0761 | A964_0643 | 1012 | —CT/TCT | L/S | indel |
| SAK_0763 | A964_0644 | 335 | CTA/CCA | L/P | non-synonymous |
| SAK_0764 | A964_0645 | — |  |  |  |
| SAK_0765 | A964_0646 | — |  |  |  |
| SAK_0785 | A964_0653 | — |  |  |  |
| SAK_0786 | A964_0654 | — |  |  |  |
| SAK_0787 | A964_0655 | — |  |  |  |
| SAK_0788 | A964_0656 | — |  |  |  |
| SAK_0789 | A964_0657 | — |  |  |  |
| SAK_0790 | A964_0658 | — |  |  |  |
| SAK_0791 | A964_0659 | — |  |  |  |
| SAK_0792 | A964_0660 | — |  |  |  |
| SAK_0793 | A964_0661 | — |  |  |  |
| SAK_0794 | A964_0662 | — |  |  |  |

—: no matched result.
